# *Phakopsora euvitis* Causes Unusual Damage to Leaves and Modifies Carbohydrate Metabolism in Grapevine

**DOI:** 10.3389/fpls.2017.01675

**Published:** 2017-09-26

**Authors:** Antonio F. Nogueira Júnior, Rafael V. Ribeiro, Beatriz Appezzato-da-Glória, Marli K. M. Soares, Júlia B. Rasera, Lilian Amorim

**Affiliations:** ^1^Department of Epidemiology, Plant Pathology, ESALQ, University of Sao Paulo, Piracicaba, Brazil; ^2^Department of Plant Biology, Institute of Biology, University of Campinas, Campinas, Brazil

**Keywords:** carbohydrate metabolism, gas exchange, grapevine rust, *Phakopsora euvitis*, photosynthetic limitations, starch accumulation, *Vitis labrusca*

## Abstract

Asian grapevine rust (*Phakopsora euvitis*) is a serious disease, which causes severe leaf necrosis and early plant defoliation. These symptoms are unusual for a strict biotrophic pathogen. This work was performed to quantify the effects of *P. euvitis* on photosynthesis, carbohydrates, and biomass accumulation of grapevine. The reduction in photosynthetic efficiency of the green leaf tissue surrounding the lesions was quantified using the virtual lesion concept (*β* parameter). Gas exchange and responses of CO_2_ assimilation to increasing intercellular CO_2_ concentration were analyzed. Histopathological analyses and quantification of starch were also performed on diseased leaves. Biomass and carbohydrate accumulation were quantified in different organs of diseased and healthy plants. Rust reduced the photosynthetic rate, and *β* was estimated at 5.78, indicating a large virtual lesion. Mesophyll conductance, maximum rubisco carboxylation rate, and regeneration of ribulose-1,5-bisphosphate dependent on electron transport rate were reduced, causing diffusive and biochemical limitations to photosynthesis. Hypertrophy, chloroplast degeneration of mesophyll cells, and starch accumulation in cells close to lesions were observed. Root carbohydrate concentration was reduced, even at low rust severity. Asian grapevine rust dramatically reduced photosynthesis and altered the dynamics of production and accumulation of carbohydrates, unlike strict biotrophic pathogens. The reduction in carbohydrate reserves in roots would support polyetic damage on grapevine, caused by a polycyclic disease.

## Introduction

Rusts have always been considered important threats to crops due to the damage they incite in the host plant. Causal agents of rusts are biotrophic pathogens which have highly developed infection structures, such as, haustoria, and limited lytic enzymes. In addition these pathogens induce long-term suppression in host defense (Mendgen and Hahn, 2002). Although they are highly specialized parasites, rust pathogens reduce host growth by diverting photoassimilates to their own development and reducing the host photosynthetic area (Helfer, 2014). At the turn of the twenty-first century, many rusts emerged or re-emerged in different plants such as, wheat (Line, 2002; Hodson, 2011), soybean (Yorinori et al., 2005; Sikora et al., 2014; Murithi et al., 2016), coffee (Avelino et al., 2015), Myrtaceae species (Morin et al., 2012), sugarcane (Zhao et al., 2011), and grapevine (Vida and Tessmann, 2005; Primiano et al., 2017). Climate change, adaptability of rust pathogens to changing conditions, and exponential growth in air travel and international trade are possible causes for rust emergence (Hodson, 2011). Recent rust epidemics in wheat, coffee and soybean caused negative economic (Hodson, 2011; Sikora et al., 2014), social (Avelino et al., 2015), and environmental (Godoy et al., 2016) impacts around the world.

Asian grapevine rust, henceforth called grapevine rust, is an endemic disease in Asia, where it was first described in 1895 (Ono, 2000). In 2001, the disease was reported in northern Australia, which is more than 3,000 km distant from the Australian grapevine commercial areas. An eradication programme started in 2001 through annual surveys to eliminate infected plants. In July 2007, northern Australia was free from *P. euvitis* and the disease was considered eradicated (EPPO—European Mediterranean Plant Protection Organization, 2007). The disease was also detected in Brazil in 2001; however, unlike Australia, the disease spread over a radius of 1,000 km in a 5-year period, and it is now considered endemic in the country. The cultivation of grapevines increased after 1960 in Brazil, when cultivars produced by national breeding programmes and adapted to tropical and subtropical climates began to be distributed to growers. Currently, grapevines in Brazil cover an area of 77,544 ha (IBGE – Instituto Brasileiro de Geografia e Estatística., 2016), 50% of which is occupied by table grape cultivars for fresh fruit and juice production. Brazilian breeding programmes for table grape and juice production are based on interspecific crossings, mainly with the use of *Vitis labrusca*, which is highly susceptible to grapevine rust (Camargo, 2004).

Initial symptoms of grapevine rust are small, circular, yellow-orange pustules scattered on the lower (abaxial) surface of the leaf. The pustules enlarge to approximately 5 mm^2^, becoming irregular and necrotic. On the upper (adaxial) side of the leaves, the lesions have an angular shape. Under high disease severity, the lesions are densely distributed, and premature defoliation occurs. Dispersal of urediniospores occurs mainly by wind. High humidity and darkness enhances urediniospore germination (Edwards, 2015). The latent period ranges from 6 to 13 days on cultivar Niagara Rosada (*V. labrusca*) depending on the temperature (Angelotti et al., 2014).

Usually, the impact of rust pathogens on CO_2_ assimilation is limited to the lesioned area invaded by fungus mycelia (Scholes and Rolfe, 1996). However, necrotrophic pathogens can reduce CO_2_ assimilation on asymptomatic areas of diseased leaves as well. This phenomenon is called “virtual lesion,” which corresponds to the leaf area where photosynthesis is null. A virtual lesion can have the same size as or be larger than the visual lesion. The relationship between visual and virtual lesions can be assessed with the *β* parameter proposed by Bastiaans (1991). *β*-values are determined experimentally by relating the photosynthesis of leaves with different levels of visual lesions and photosynthesis in healthy leaves. Estimated *β*-values >1 indicate the existence of virtual lesions (Bastiaans, 1991). High values of virtual lesions have been found for necrotrophic/hemibiotrophic pathogens such as, 3.74 for *Pyricularia oryzae* on rice (Bastiaans, 1991), 12.12 for *Mycosphaerella pinodes* on dried pea (Garry et al., 1998), and 7.97 for *Colletotrichum lindemuthianum* on common bean (Bassanezi et al., 2001). Rust pathogens usually do not induce virtual lesions, or they induce only small ones (Bastiaans, 1991; Bassanezi et al., 2001).

Biotrophic pathogens also manipulate plant carbohydrate metabolism, driving plant sugars to their own needs, like a physiological sink absorbing assimilates (Zadoks and Schein, 1979). In some plant-biotrophic pathogen interactions, the leaf starch dynamics is altered. Grapevine infected by *Plasmopara viticola* shows unusually high starch accumulation at the end of the dark period in lesion regions (Gamm et al., 2011). Changes in the accumulation of carbohydrates in storage organs are also commonly observed in diseased plants (Boote et al., 1983). Carbohydrate accumulation in roots during overwintering is essential for the development of perennial plants in the following season. Reduction of carbohydrate reserves in grapevine roots was associated with a reduction of 50% in inflorescences and flowers in the subsequent year (Bennett, 2002).

*Phakopsora* rusts seem to differ from other rusts in several aspects. Many *Phakopsora* species can penetrate directly into the host (Bonde et al., 1976; Adendorff and Rijkenberg, 2000; Seier et al., 2009). Most of these pathogens cause intensive defoliation and some, such as, *P. pachyrhizi* and *P. jatrophicola*, cause extensive tissue necrosis (Melching et al., 1979; Seier et al., 2009). Damage caused by *Phakopsora euvitis* in grapevine has not been quantitatively assessed; therefore, the aim of this work was to quantify the effects of the disease on photosynthetic rates and carbohydrate dynamics. The approach used included histopathological analyses and the assessment of relationships between disease severity and photosynthesis to determine the presence of virtual lesions (*sensu* Bastiaans), revealing the underlying processes related to low photosynthetic performance, i.e., limitations imposed by rust and how root reserves are affected.

## Materials and methods

### Plant material

In all experiments, potted grapevines (*V. labrusca*) cv. Niagara Rosada grafted on IAC-766 [*Vitis riparia* × (*Vitis cordifolia* × *Vitis rupestris*) × *Vitis caribaea*] rootstocks were used. The plants were grown in pots (7 L) containing sterilized substrate (clay soil, manure and, sand at a ratio of 1:1:1) and kept in a greenhouse at an average air temperature of 25°C. After bud break, the plants were reduced to a single stem, and each pot received 200 mL of water daily. The plants were fertilized monthly with 20 g of NPK (4:14:8) fertilizer.

### Inoculum of *Phakopsora euvitis* and inoculation procedures

Grapevine diseased leaves containing urediniospores of *P. euvitis* were immersed in 200 mL of distilled water and leaves abaxial surface were scraped with a brush. Spore concentration was determined using a hemocytometer and adjusted to 10^5^ urediniospores mL^−1^. Serial dilutions were performed to obtain 10^4^, 10^3^ and 10^2^ urediniospores mL^−1^. Urediniospore suspensions of *P. euvitis* were sprayed on the abaxial leaf surface until run-off. Inoculated plants were kept in a dark inoculation room for 24 h under 100% RH and 25°C. A humidifier was used to reach 100% RH in the inoculation room. Maintenance of the inoculum was carried out through re-inoculation of urediniospores onto healthy plants every month. This method of inoculation was used in all experiments. Plants in the control treatment were sprayed with water and kept under the same conditions. In all experiments, the inoculations were performed 1 month after bud break, when plants had seven fully expanded leaves.

### Relationship between disease severity and leaf gas exchange

Two experiments were performed in a growth chamber (Conviron, Winnipeg, Canada) to determine how rust affects gas exchange of grapevine leaves. The conditions in the growth chamber were 25°C (± 2°C) with a photoperiod of 12 h, with photosynthetic active radiation (PAR) of 400 μmol m^−2^ s^−1^. Five treatments were applied in the first experiment: inoculations with 0, 10^2^, 10^3^, 10^4^ and 10^5^ urediniospores of *P. euvitis* mL^−1^. In the second experiment, three treatments were applied: inoculation with 0, $10_{,}^{3}$ and 10^5^ urediniospores of *P. euvitis* mL^−1^. The fourth fully expanded leaf (from the plant base) was used for evaluation of gas exchange in both experiments. Five and ten replications were used in the first and second experiments, respectively. The experimental design was completely randomized.

Different levels of inoculum were used to obtain a wide range of levels of disease severity. The evaluations were carried out twice a week on the same leaf area of 2 cm^2^. The net CO_2_ assimilation (*A*), stomatal conductance (*g*_s_), intercellular CO_2_ concentration (*C*i), and transpiration (*E*) were estimated in diseased and healthy leaves using a portable infrared gas analyser (LI-6400XT, LI-COR Inc., Lincoln, NE, USA). The air CO_2_ concentration (*C*a) during the measurements was 400 μmol mol^−1^, and gas exchange was measured under PAR of 800 μmol m^−2^ s^−1^. The leaf areas selected for evaluations of gas exchange were photographed, and digital images were processed with Quant software (Vale et al., 2001) to estimate the disease severity in each assessment. The pustules and the eventual yellowish or brown halo that surrounded the pustules were considered the diseased area.

### Estimating virtual lesions (*β*) using the Bastiaans model

Disease severity on the leaf areas assessed for gas exchange was related to the relative CO_2_ assimilation rate by non-linear regression according to the model:

(1) $$\begin{array}{r} P\text{x}/P\text{o}={\left(1-x\right)}^{\beta } \end{array}$$

where *P*x is the net photosynthetic rate of a leaf with rust severity *x*, and *P*o is the average photosynthetic rate of healthy leaves. The *β*-value corresponds to the effect of disease severity on the green leaf area adjacent to the lesion. The data from gas exchange experiments described in the previous section were used to estimate the *β*-values.

Values of *g*_s_, *C*i, and *E* were transformed in proportions relative to the average value of each variable in healthy leaves (*g*_s_x/*g*_s_o, *C*ix/*C*io, and *E*x/*E*o) in both experiments. Bastiaans model (Equation 1) was also used to fit the data of *g*_s_x/*g*_s_o and *E*x/*E*o (dependent variables) vs. disease severity (independent variable). Linear regressions were performed between relative *C*i and disease severity. Statistica 6.0 software was used to perform non-linear regressions.

### Photosynthetic limitations of plants infected with *Phakopsora euvitis*

Two experiments were carried out to determine the photosynthetic limitations of plants infected with *P. euvitis*. Three plants were inoculated with *P. euvitis* at a concentration of 10^4^ urediniospores mL^−1^, and three plants were sprayed with water. After inoculation, plants were kept at greenhouse at temperature of 25°C (± 2°C). Six evaluations were made, two per day, beginning on the 15th day after inoculation. The evaluations were carried out with a LI-6400XT equipped with a fluorimeter (6400-40, LI-COR Inc., Lincoln, NE, USA). Some photosynthetic variables were estimated from curves of photosynthesis response to increasing chloroplastic CO_2_ concentration.

Photosynthesis measurements were begun with *C*a of 400 μmol mol^−1^ that was gradually reduced (250, 150, 100) to 50 μmol mol^−1^ and then gradually increased (400, 600, 800, 1,400) up to 2,000 μmol mol^−1^. Respiration (*R*) represents the intercept of the linear regression from initial values of the *A*/*C*i curve. Mesophyll conductance (*g*_m_) was calculated as:

(2) $$\begin{array}{r} g_{\text{m}}=A/\left(C\text{i}-\left(\Gamma^{*}\left(J+8\left(A+R\right)\right)\right)/\left(J-4\left(A+R\right)\right)\right) \end{array}$$

where Γ* is the photosynthetic compensation point, i.e., the CO_2_ concentration at which the photorespiratory efflux of CO_2_ is equal to the CO_2_ photosynthetic assimilation rate; and *J* is the transport of electrons from chlorophyll fluorescence assessments. The CO_2_ concentration at the site of carboxylation in the chloroplast (*C*c) was obtained with the following equation:

(3) $$\begin{array}{r} C\text{c}=C\text{i}-A/g_{\text{m}} \end{array}$$

A/Cc curves were obtained and V_cmax_ and J_max_ were estimated (Farquhar et al., 1980; Sharkey et al., 2007; Flexas et al., 2008):

(4) $$\begin{array}{l} A\;=V_{\text{cmax}}\left(Cc-\Gamma^{*}\right)/\left(Cc+K_{c}\left((1-\left(O/K_{o}\right)\right)\right) \end{array}$$

(5) $$\begin{array}{r} A=J_{\text{max}}\left(Cc-\Gamma^{*}\right)/4\left(Cc+2\Gamma^{*}\right) \end{array}$$

where *K*_c_ and *K*_o_ are the Michaelis-Menten constants of Rubisco for carboxylation and oxygenation, respectively, and *O* is the internal O_2_ concentration, considered equal to the external O_2_ concentration (Flexas et al., 2007). *V*_cmax_ and *J*_max_ were estimated by non-linear regressions with software STATISTICA 6.0.

From *A*/*C*c curves, the *A*_max_ and *g*_smax_ parameters that corresponded to the maximum amount of CO_2_ assimilation and the maximum stomatal conductance, respectively, were also obtained. Stomatal limitation (*L*s) was calculated considering the *A*-values at *C*a of 400 μmol mol^−1^ and at *C*i of 400 μmol mol^−1^ (Farquhar and Sharkey, 1982). The average values of photosynthesis-related variables of healthy and diseased plants were compared by Student's *t*-test (*P* ≤ 0.05).

### Histopathological analyses

Two experiments were carried out in a greenhouse for the histopathological analyses of *P. euvitis* in cv. Niagara Rosada. Seven plants were inoculated with urediniospore suspensions of *P. euvitis* at a concentration of 10^4^ urediniospores mL^−1^, and seven plants were sprayed with water.

Five diseased leaves and five healthy leaves were sampled at dawn of the 14th and 40th days after inoculation for light microscopy analyses. Inoculated and healthy samples were fixed in Karnovsky solution modified with phosphate buffer pH 7.2. During fixation, a vacuum pump was used to remove air from the tissues, and then samples were dehydrated in an ethanol series (10, 20, 30, 40, 50, 60, 70, 80, 90, and 100%) and infiltrated in hydroxyethyl methacrylate (Leica Historesin®), as recommended by the manufacturer. The blocks were sectioned at 5–7 μm thick in a rotational microtome (Leica RM 2045) with the aid of a steel blade (type C). Next, the slides were deposited on a hot plate at 40°C to dry and fix the sections on the blade. To detect starch grains, zinc-chloride iodine (Strasburger, 1913) was used on the sections on the blades that were mounted on the reagent itself. Anatomy images were taken by using a Leica® DC video camera attached to a Leica® microscope 300F (Leica Microsystems Heerbrugg GmbH, Heerbrugg, Switzerland).

An area of 25 cm^2^ was removed from the same leaves used for anatomy to determine the starch content. The samples were dried with forced air circulation (65°C) until they reached constant weight. Subsequently, the starch contents in the leaf fragments were determined using the enzymatic method proposed by Amaral et al. (2007). The contents of starch in healthy and diseased leaves were compared by Student's *t*-test (*P* ≤ 0.05).

Samples of diseased leaves were collected 22 days after inoculation and immediately fixed in a buffer containing 3% glutaraldehyde and 0.2 M cacodylate, pH 7.25, for transmission electron microscopy analyses. The samples were air-dried in a vacuum chamber for 5 min, post-fixed in 1% osmium tetroxide for 2 h, dehydrated through a graded series of acetone solutions (30, 50, 70, 90, and 100%), and embedded in Spurr's resin. The blocks were sliced with a Leica UC6 ultramicrotome. The sections were treated using 5% uranyl acetate and 2% lead citrate for 30 min each for contrast (Reynolds, 1963). Electron micrographs were taken using a Gatan 830.J46W44 video camera attached to a Jeol transmission electron microscope (JEM-1011) at an acceleration voltage of 60 kV.

### Dry matter partitioning and carbohydrate contents in grapevine

Two experiments were conducted, with five treatments: inoculation with 0, 10^2^, 10^3^, 10^4^, and 10^5^ urediniospores mL^−1^ of *P. euvitis*. The experimental design was completely randomized with five replications. The plants were kept in a greenhouse for 45 days after inoculation. Rust severity (Angelotti et al., 2008) and leaf area (Li-3100, LI-COR Inc., Lincoln, NE, USA) were estimated for all leaves of each plant. The stems, trunk and roots of each plant were collected and dried in an oven with forced air circulation (65°C) until they reached constant weight. The concentrations and total contents (concentration * dry mass) of carbohydrates in roots were determined. Soluble carbohydrates were extracted with a methanol:chloroform:water (MCW) solution, according to Bieleski and Turner (1966). The starch concentration was quantified in 10-mg samples from the insoluble fraction obtained from the soluble carbohydrate extraction. The contents of sucrose, total soluble sugars and starch were determined according to Dubois et al. (1956), van Handel (1968) and Amaral et al. (2007), respectively.

Linear regressions were performed on the relationships between leaf area and root biomass (dependent variables) and disease severity (independent variable). The relationships between the proportion of sucrose and starch contents (relative to the average of healthy plants) and disease severity were described by the power model

(6) $$\begin{array}{r} y={ax}^{-b} \end{array}$$

where *y* is the relative carbohydrate content, *x* is disease severity and *a* and *b* are function parameters. The relationship between relative total soluble sugar content and disease severity was described by a negative exponential model

(7) $$\begin{array}{r} y=a\exp\left(-bx\right) \end{array}$$

where *y* is the relative sugar content, *x* is disease severity and *a* and *b* are function parameters. Statistica 6.0 software (StatSoft Inc., Tulsa, OK, USA) was used in all statistical analyses (Equation 7). Non-linear regressions were performed with STATISTICA 6.0 software.

## Results

### Relationship between disease severity and leaf gas exchange

**Figure 1:**
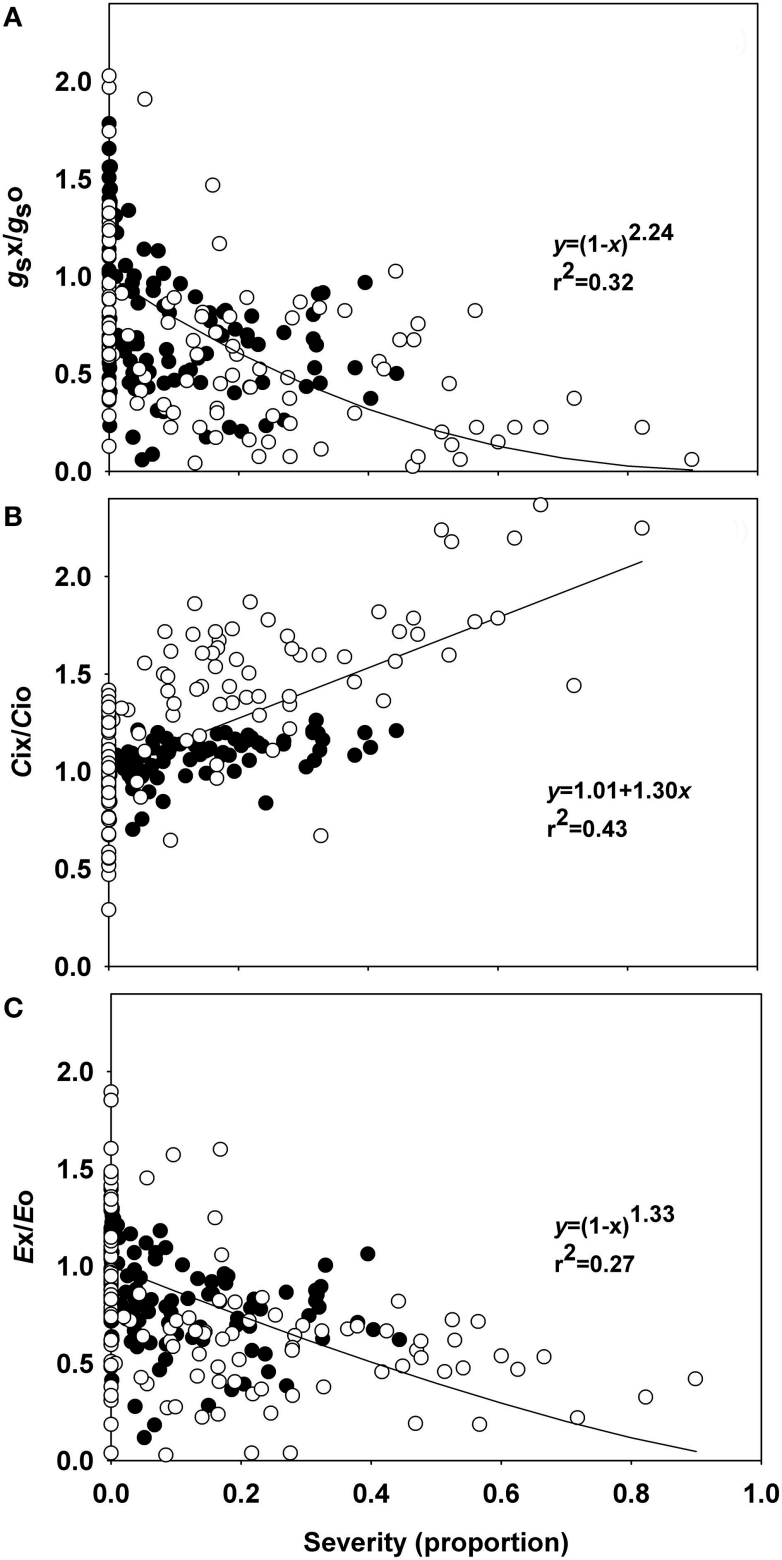
Relationships between the relative stomatal conductance (*g*_s_), intercellular CO_2_ concentration (*C*i), transpiration (*E*), and rust severity (*Phakopsora euvitis*) in grapevine cv. Niagara Rosada. The black circles represent data from the first experiment, and the white circles represent data from the second experiment. Lines represent Bastiaans model in **(A,C)** and the linear model in **(B)**. The average values for *g*_s_, *C*i, and *E* in healthy leaves of experiment 1 are, respectively, 0.34 mol H_2_O m^−2^ s^−1^, 301.87 μmol CO_2_ mol^−1^, and 6.82 mmol H_2_O m^−2^ s^−1^. The average values for *g*_s_, *C*i, and *E* in healthy leaves of experiment 2 are, respectively, 0.13 mol H_2_O m^−2^ s^−1^, 216.62 μmol CO_2_ mol^−1^, and 2.09 mmol H_2_O m^−2^ s^−1^.

The incubation period for *P. euvitis* was 7 days in all experiments. Rust severity reached 45 and 90% in experiments 1 and 2, respectively (Figure 1, Supplementary Figure 1). The *A*-values in healthy plants ranged from 8 to 15 μmol m^−2^ s^−1^, with an average of 12 μmol m^−2^ s^−1^. The *g*_s_-values in healthy plants ranged from 0.06 to 0.61 mol m^−2^ s^−1^. The average values of *C*i and *E* in healthy plants were, respectively, 245.6 μmol mol^−1^ and 3.72 mmol m^−2^ s^−1^. High variability in the relationships between disease severity vs. *g*s, *C*i, and *E* were observed (Figure 1). All regressions were significant, though the coefficients of determination were below 0.5. Slight decreases in relative values of g_*s*_ (*g*_s_x/*g*_s_o) and *E* (*E*x/*E*o) were noticed when the severity of rust increased (Figures 1A,C). Both variables decreased according to equation 1 that estimated reductions for *g*s and *E* of 97 and 88%, respectively, for rust severity of 80% (Figures 1A,C). Relative *C*i (*C*i/*C*o) was directly proportional to disease severity, and an increase of 1.3% in disease severity corresponded to a 1% increase in intercellular CO_2_ concentration (Figure 1B).

### Estimates of virtual lesions (*β*) using the model of Bastiaans

**Figure 2:**
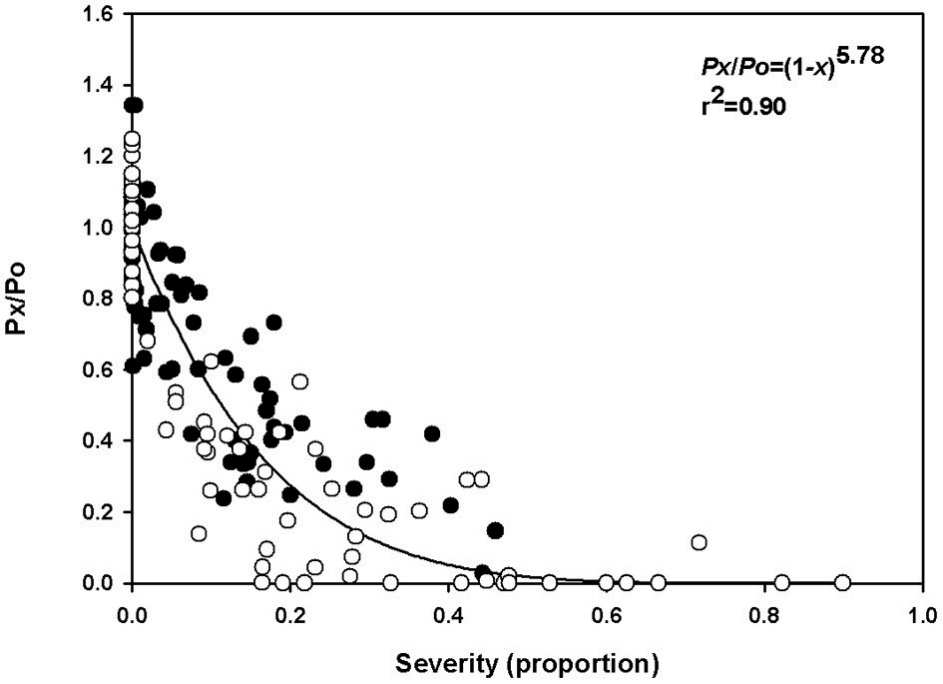
Relationship between the relative net photosynthesis rate (*P*x/*P*o) and rust severity (*Phakopsora euvitis*) in grapevine cv. Niagara Rosada. The black circles represent data from the first experiment, and the white circles represent data from the second experiment (Total *n* = 158). The line represents the model *y* = (1 − *x*)^β^.

A steep decrease in the relative net photosynthetic rate was observed even at low rust severity, and null values of relative photosynthesis were observed in severities higher than 40% in experiment 1 and over 20% in experiment 2 (Figure 2). Low variability was observed in the relationships between disease severity and relative net photosynthetic rate, which was reflected by the high coefficient of determination (*r*^2^ = 0.90 and standard error = 0.35). The value of the *β* parameter, estimated by the model of Bastiaans, was 5.78. The estimated reduction of the net photosynthetic rate was 46 and 73% for disease severities of 10 and 20%, respectively. According to the model, the relative net photosynthetic rate becomes zero when rust severity reaches 40%.

### Photosynthesis limitations in plants infected with *Phakopsora euvitis*

Plants infected with *P. euvitis* showed lower photosynthetic activity than healthy plants. *V*_cmax_, *J*_max_, *g*_m_, *g*_smax_, and *A*_max_ were significantly higher in healthy plants than in diseased ones (Table 1). The reduction in these parameters in diseased plants (average of disease severity = 7.2%) ranged from 35 to 67%, and g_m_ was the photosynthetic variable most affected by rust. There was no significant difference in *L*s between healthy and diseased plants (Table 1).

**Table 1:** Maximum Rubisco carboxylation rate (*V*_cmax_), maximum rate of electron transport driving regeneration of ribulose-1,5-bisphosphate (*J*_max_), mesophyll conductance (*g*_m_), maximum stomatal conductance (*g*_smax_), stomatal limitation (*L*_s_), and maximum rate of net photosynthesis (*A*_max_) in grapevine plants affected by rust (*Phakopsora euvitis*).

**Variable**	**Healthy plants**		**Infected plants**^a^	
*V*_cmax_ (μmol m^−2^ s^−1^)	194.4	a	101.0	b
*J*_max_ (μmol m^−2^ s^−1^)	170.5	a	109.1	b
*g*_m_ (mol m^−2^ s^−1^)	0.15	a	0.05	b
g_smax_ (mol m^−2^ s^−1^)	0.33	a	0.15	b
*L*s (%)	14.6		20.9	ns
*A*_max_ (μmol m^−2^ s^−1^)	28.0	a	16.9	b

*Values followed by the different letter in the row are significantly different by the Student t-test (P ≤ 0.05), ns means not significantly different*.

a *Mean severity (± standard deviation) of rust in diseased plants was 7.2 ± 4.2%*.

### Histopathological analyses

**Figure 3:**
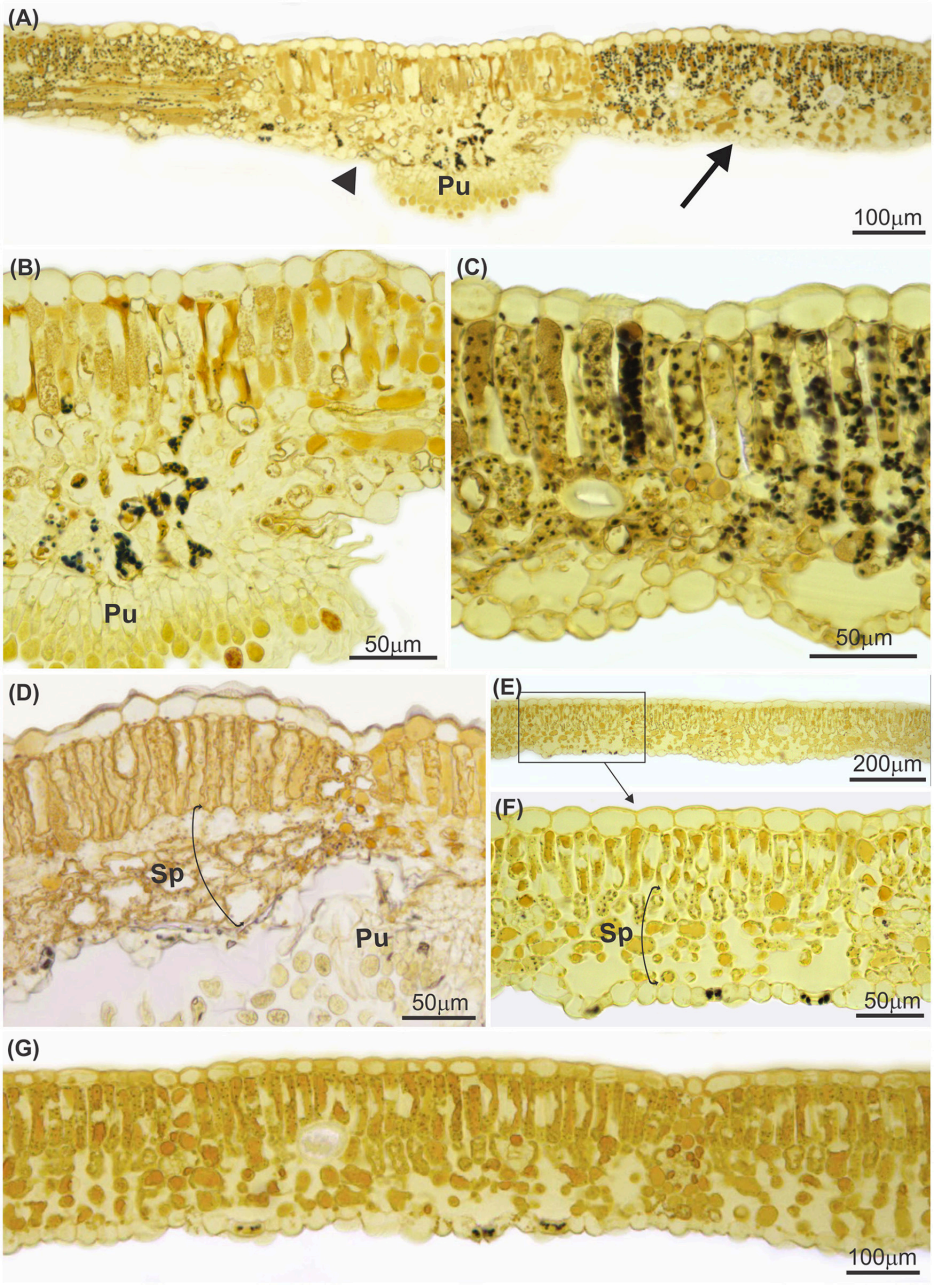
Cross sections of leaves of *Vitis labrusca* cv. Niagara Rosada infected with *Phakopsora euvitis*, 14 **(A–C)** and 40 **(D)** days after inoculation and healthy leaves **(E–G)**. In **(A)**, note the almost absence of starch in the mesophyll underlying the pustule of *P. euvitis* (arrowhead) and the significant starch accumulation in the mesophyll in regions adjacent to pustules (arrow). These two leaf sectors are in detail in **(B,C)**, respectively. Starch accumulation is not observed in the healthy mesophyll **(E–G)**. Spongy parenchyma cells hypertrophied in the regions near the pustules in leaves **(D)** and the spongy parenchyma with intercellular spaces in the healthy tissue **(E–G)**. Pu, pustule; Sp, spongy parenchyma.

**Figure 4:**
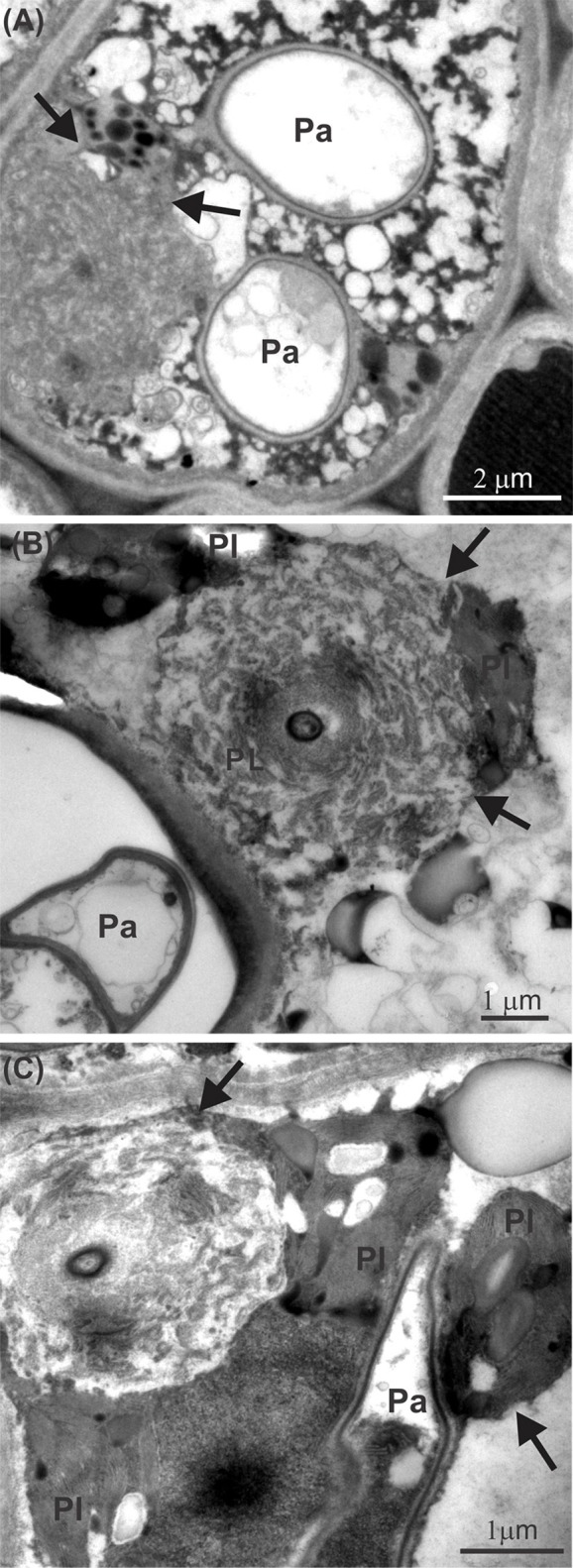
Transmission electron micrographs of leaves of *Vitis labrusca* cv. Niagara Rosada infected with *Phakopsora euvitis* showing chloroplast degeneration (arrows) in the spongy parenchyma cells **(A–C)**. Ch, chloroplast; Pa, pathogen haustoria.

Leaves infected with *P. euvitis* showed low starch contents in the mesophyll underlying the pustules (Figures 3A,B). However, there was a significant increase in the accumulation of starch in the chlorophyll parenchyma in regions adjacent to pustules (Figures 3A,C) compared to healthy leaves (Figures 3E–G). The starch concentration was significantly higher (*P* = 0.02) in diseased (33.1 mg g^−1^) than in healthy (22.9 mg g^−1^) leaves, which corresponds to an increase of 44% in starch concentration in leaves with rust symptoms. The spongy parenchyma of diseased leaves showed reduction of intercellular spaces due to cell hypertrophy surrounding the pustules (Figure 3D) when compared with the spongy parenchyma of healthy leaves (Figures 3E–G). Chloroplast degeneration due to disruption of plastid membranes was observed in chlorophyll parenchyma cells infected with *P. euvitis* (Figure 4). All degenerated chloroplasts were close to the pathogen haustoria (Figure 4).

### Biomass and carbohydrates as affected by rust

**Figure 5:**
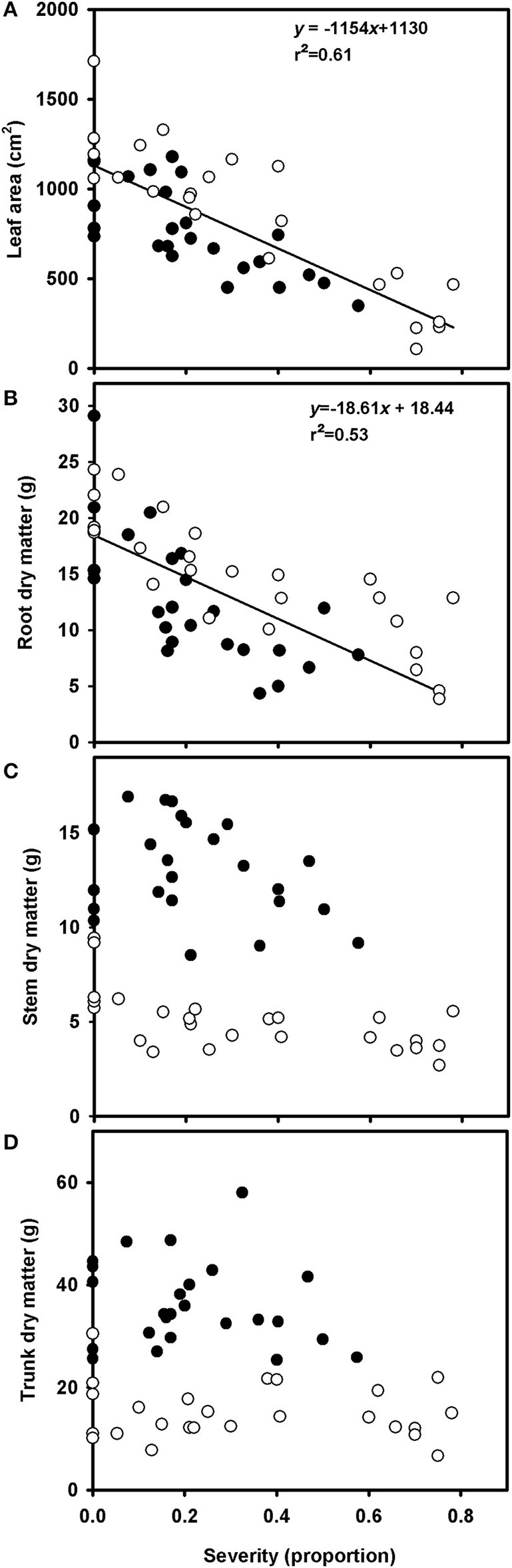
Relationships between leaf area **(A)**, root dry matter **(B)**, trunk dry matter **(C)**, and stem dry matter **(D)** of grapevine cv. Niagara Rosada grafted on IAC 766 and rust severity (*Phakopsora euvitis*). The black circles represent data from the first experiment, and the white circles represent data from the second experiment. Lines represent the linear regressions (*P* < 0.05).

Total leaf area of healthy plants ranged from 735 to 1712 cm^2^. Total leaf area decreased linearly with increasing rust severity, due to defoliation (Figure 5A, Supplementary Figure 2). According to the linear model, an increase of 1% in disease severity accounts for 11% reduction in leaf area. A linear reduction in root dry matter with increasing rust severity was also observed, although the variability was higher than that in the relationship between disease severity and leaf area (Figure 5B, Supplementary Figure 3). At 80% disease severity, the root dry matter decreased from 18.51 to 4.11 g. No correlation was found between disease severity and dry matter of trunks or stems in grapevine (Figures 5C,D).

**Figure 6:**
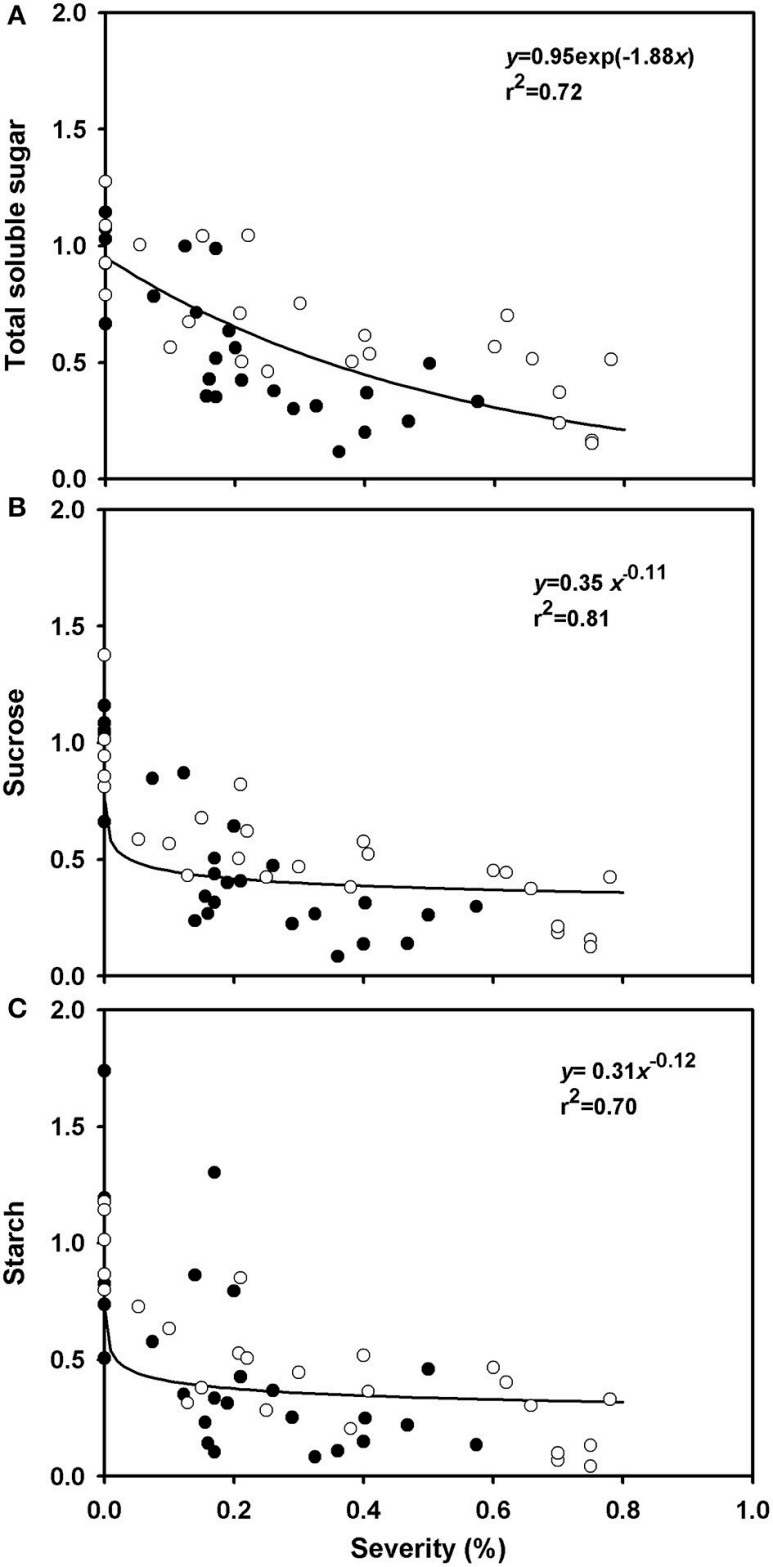
Relative variations of total soluble sugars (AST), sucrose and starch in roots of grapevine cv. Niagara Rosada as a function of rust severity (*Phakopsora euvitis*) 45 days after inoculation. Data from the dependent variables were compared to the average data of control plants. Open circles correspond to the data of experiment 1 and closed circles, data from experiment 2. Lines represent the exponential negative **(A)** and power models **(B,C)**.

The average concentrations of total soluble sugars, sucrose, and starch in healthy plants were, respectively, 55.4, 43.2, and 118 mg.g^−1^ of root dry matter, and the average contents of total soluble sugars, sucrose and starch were, respectively, 773.3, 659, and 2,850 mg per root (data not shown). The relative contents of these carbohydrates in roots decreased with increasing rust severity (Figure 6). The availability of sucrose in diseased plants (rust severity higher than 20%) was half the sucrose availability in healthy plants (Figure 6B). A reduction of 60% in starch availability was observed in plants with rust severities between 20 and 80% (Figure 6C).

## Discussion

Colonization by *P. euvitis* caused a significant reduction in CO_2_ assimilation rate in leaves of *V. labrusca*. The impairment of the photosynthetic rate of leaves was caused by the effects of the pathogen on the green symptomless tissues surrounding pustules, which was quantified by the *β* parameter (5.78). *β*-values higher than 2 are unusual for rusts. In the pathosystems *Puccinia recondita* f. sp. *Tritici*—wheat (Spitters et al., 1990; Bastiaans, 1991), *Uromyces appendiculatus*—common bean (Bassanezi et al., 2001), and *Puccinia triticina*—wheat (Robert et al., 2005), *β*-values were 1.2, 2.1, and 1.0, respectively. Even for *Phakopsora parchyrhizi*, which causes severe damage in soybean, *β*-values ranged from 2.1 to 2.5 (Kumudini et al., 2010). Low disturbance of colonized tissue is expected in plants infected by biotrophic pathogens, as evolutionarily, a functional host is advantageous and necessary for their survival (Shtienberg, 1992). However, *P. euvitis* in grapevine does not fit this generalization. The damage caused by *P. euvitis* has a magnitude of damage similar to that caused by necrotrophic or hemibiotrophic pathogens. *β*-values quantified in the pathosystems *Magnaporthe oryzae—*rice, *Pseudocercospora griseola*—common bean, and *C. lindemuthianum*—common bean were 3.0–3.7, 3.8, and 7.9, respectively. High values for the *β* parameter have also been reported for some rusts; however, in those cases, disease severity was underestimated because the halos surrounding uredia were disregarded (McGrath and Pennypacker, 1990; Robert et al., 2005). In our study, the leaf tissue adjacent to lesions showed several changes, such as, hypertrophy of mesophyll cells, decrease in intercellular space, and chloroplast degeneration. In addition, in the green leaf tissue surrounding the lesions, over a distance that was twice the lesion size, mesophyll cells could show unusual starch accumulation. These important changes in mesophyll tissue can explain the high *β*-value observed in this study.

*U. appendiculatus* in common bean and *Puccinia hordei* in barley plants also induce starch accumulation in the chloroplasts of colonized cells or their neighbors, but rarely in cells beyond the pustule edge (Sziráki et al., 1984; Scholes and Farrar, 1987). Starch accumulation may be the result of increased activity of acid invertase, which hydrolyses sucrose in hexoses in the infection sites of rusts (Long et al., 1975). As the chloroplasts of the cells surrounding pustules are damaged, starch synthesis occurs in the first available chloroplasts (Long et al., 1975; Hay and Walker, 1989). Starch accumulation due to increased acid invertase activity in tissues surrounding pustules would cause the down-regulation of the Calvin cycle and the reduction of Rubisco activity (Wagner and Boyle, 1995; Bassanezi et al., 2002). The reduction of Rubisco activity observed in this study has been reported in other biotrophic pathosystems (Ayres, 1981; Scholes et al., 1994; Bassanezi et al., 2000). However, the magnitude of the reduction in Rubisco activity in diseased *Vitis* was much higher (47%, Table 1) than that of other rusts, such as, common bean rust (27%, Bassanezi et al., 2000). Leaves of beans with angular leaf spot (*P. griseola*) showed similar reductions in Rubisco activity, in the range of 30–47% (Bassanezi et al., 2000). The reduction in regeneration of RuBP dependent on electron transport (Table 1) in infected leaves of grapevine can be related to the rupture of chloroplast membranes with release of their contents to the cytoplasm (Figure 4) in mesophyll regions adjacent to the pustules.

The impressive reduction in the diffusion of CO_2_ in the mesophyll (*g*_m_) observed in this work could be a consequence of a reduction in the efficiency of the CO_2_ fixation process (Gordon and Duniway, 1982) and of physical barriers to CO_2_ diffusion (Flexas et al., 2012). The hypertrophy of parenchyma cells that was observed near pustules contributes to the reduction of intercellular spaces, which may act as a physical barrier to CO_2_ diffusion. Hypertrophy of spongy parenchyma cells and decreased intercellular spaces were also observed in leaves of soybean infected with *Phakopsora pachyrhizi* (Bonde et al., 1976). Histological and morphological changes, such as, cellular hypertrophy and galls, are associated with symptoms of microcyclic rusts. These changes can be caused by the production of growth regulatory substances by pathogens and/or by plants (Quilliam and Shattock, 2003). In the case of macrocyclic rusts, such as, those in the genus *Phakopsora*, the cause of these histopathological changes in cells surrounding infection sites remains unknown. Taking into account the changes in photosynthetic traits related to mesophyll processes (Table 1) and the differential sensitivities of stomatal conductance and photosynthesis (Figures 1A, 2), our data clearly indicate that *P. euvitis* reduces grapevine photosynthesis through non-stomatal limitation, imposing damage on CO_2_ fixation by Rubisco and RuBP regeneration through electron transport chains in the thylakoids and by reducing CO_2_ transport to carboxylation sites inside the chloroplasts.

Increased transpiration is commonly observed in diseased leaves when pustules rupture the leaf epidermis, as in graminicolous rusts (Shtienberg, 1992). However, we did not find increases in transpiration in diseased plants even under high rust severity, which agrees with the behavior of common bean rust (Bassanezi et al., 2002). Due to the slight effect of this pathogen in stomatal resistance, a clear relationship between *g*_s_ and *E* vs. disease severity was not observed. Low values of determination coefficients (*r*^2^) in the non-linear regressions reinforce the weak relationship between disease severity and *g*_s_ or *E*.

The reduction in biomass and carbohydrates in the roots of diseased plants is a consequence of colonization of *P. euvitis* in leaves, which caused an imbalance in the translocation of photoassimilates. Changes in photoassimilate translocation are attributed mainly to the establishment of new sinks in the pathogen infection sites. Both necrotrophic and biotrophic pathogens can cause disturbances in the translocation of assimilates. However, carbohydrate accumulation in infected tissues is a typical feature of biotrophic fungi (Hay and Walker, 1989). Leaves of common bean infected with *U. appendiculatus* behave as photoassimilate sinks, reducing the partitioning of carbon into roots and new leaves. Leaves of barley infected with *P. hordei* export less sucrose to other plant organs, and diseased plants show reduced growth (Owera et al., 1983). The reduction in carbohydrate accumulation in roots of *V. labrusca* is significant even in plants with low rust severity levels (Figure 6). As grapevine plants use starch stored in roots for their growth in the early stages of development from bud break until the beginning of flowering (Keller, 2010), the occurrence of grapevine rust in one season can reduce grapevine vigor in the subsequent season (Vida and Tessmann, 2005). Polyetic yield loss, i.e., an increase in yield losses from 1 year to the next, caused by a disease, is a common feature of systemic diseases in perennial crops. In these pathosystems, there is inoculum accumulation; consequently, the build-up of the epidemic takes many years (Zadoks and Schein, 1979). Grapevine rust is a polycyclic disease in which the inoculum does not accumulate over seasons. However, yield losses caused by this disease are typically polyetic. The reduction in the photosynthetic rate and carbohydrate production that is associated with defoliation caused by *P. euvitis* in one season leads to retarded bud break and flowering and to a reduction in the number of flowers and the weight of clusters in the following season (CABI - Centre for Agriculture Biosciences International, 2015). As the onset of grapevine rust frequently occurs after harvest in the tropics, the yield losses caused by the current epidemics are usually unnoticed. The polyetic losses, however, are important in the long term. A similar situation was reported for coffee diseases in Central America (Avelino et al., 2015; Cerda et al., 2017).

In conclusion, *P. euvitis* dramatically reduced photosynthesis and altered the dynamics of production and accumulation of carbohydrates in *V. labrusca*. The alternative sink for photoassimilates and the changes in translocation patterns caused by *P. euvitis* are common characteristics of biotrophic pathogens (Lewis, 1973). However, the magnitude of the reduction in the photosynthetic rate of asymptomatic tissue surrounding the lesion is comparable to that caused by necrotrophic pathogens (Bassanezi et al., 2000, 2001). The dichotomy of biotrophic and necrotrophic pathogens may give the impression that they represent absolute categories. In fact, there is a continuous gradation between the two types of pathogens (Lucas, 1998). *P. euvitis* is within this evolutionary continuum, closer to the strict biotrophic, but with some necrotrophic features in its type of colonization.

## Author contributions

AN, RR, and LA conceived, planned, and designed the research, and wrote the manuscript. AN performed the experiments involving gas exchange and carbohydrates dynamics. BA was responsible for design and interpretation of histopathological experiments. MS and JR performed the optical and electron microscopy. All authors reviewed the final version of the manuscript.

### Conflict of interest statement

The authors declare that the research was conducted in the absence of any commercial or financial relationships that could be construed as a potential conflict of interest. The reviewer MJ and handling Editor declared their shared affiliation.
