# Supplementary material for: Phakopsora euvitis Causes Unusual Damage to Leaves and Modifies Carbohydrate Metabolism in Grapevine
Source: Front Plant Sci. 2017 Sep 26;8:1675. doi: 10.3389/fpls.2017.01675 (PMC5623187; doi:10.3389/fpls.2017.01675)
Supplement: Supplementary file 1 [file DataSheet1.DOCX]

Supplementary Material

*Phakopsora euvitis* causes unusual damage to leaves and modifies carbohydrate metabolism in grapevine

Antonio F. Nogueira Jr., Rafael V. Ribeiro, Beatriz Appezzato-da-Gloria, Marli K.M. Soares, Julia B. Rasera, Lilian Amorim *

* Correspondence: Corresponding Author: lilian.amorim@usp.br

## Supplementary Figures


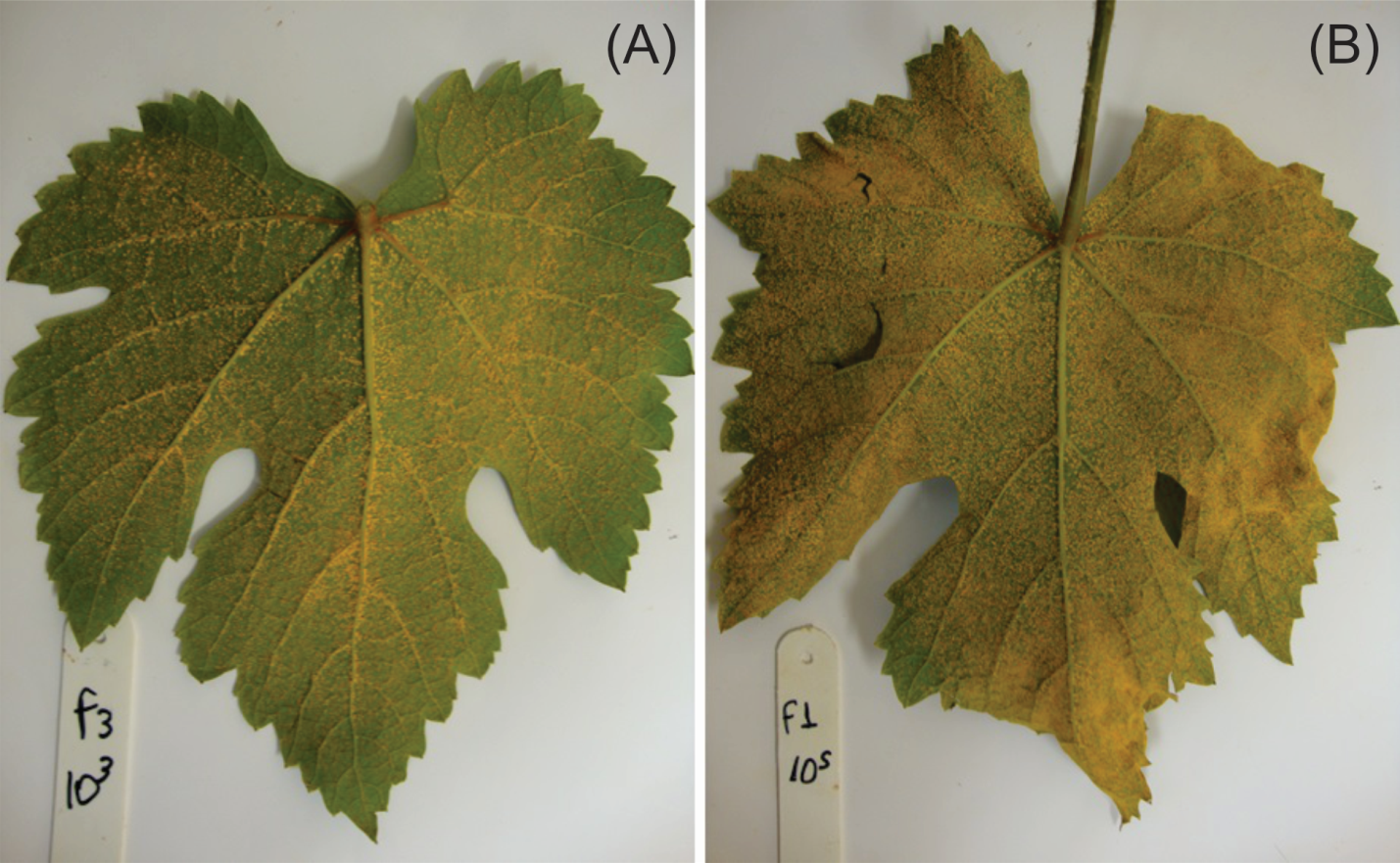


**Supplementary Figure 1.** Rust symptoms on *Vitis labrusca* cv. Niagara Rosada leaves 21 days after inoculation with 10^3^ (A) and 10^5^ (B) urediniospores of *Phakopsora euvitis* mL^-1^


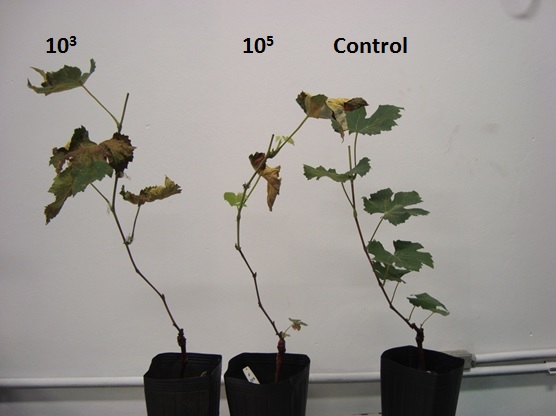


**Supplementary Figure 2.** Defoliation caused by *Phakopsora euvitis* in *Vitis labrusca* cv. Niagara Rosada 45 days after inoculation with 10^3^ and 10^5^ urediniospores of *Phakopsora euvitis* mL^-1^. Healthy (control) plant with 7 leaves at the right.


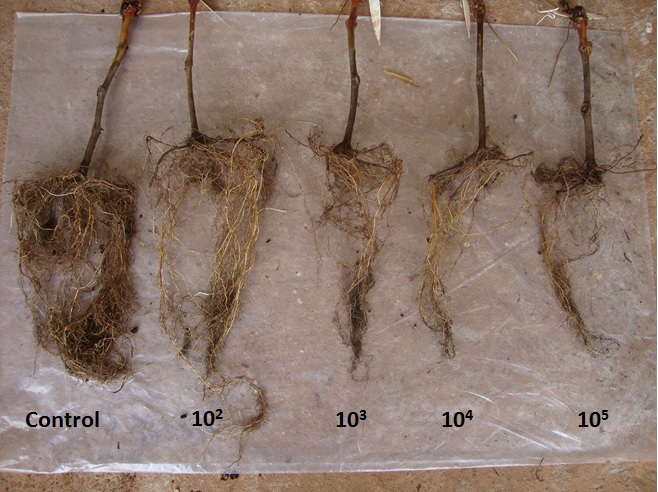


**Supplementary Figure 3.** Root systems of *Vitis labrusca* cv. Niagara Rosada 45 days after inoculation with 10^2^, 10^3^, 10^4^, and 10^5^ urediniospores of *Phakopsora euvitis* mL^-1^. Healthy (control) plant at left.
